# Supplementary material for: Complex‐centric proteome profiling by SEC‐SWATH‐MS
Source: Mol Syst Biol. 2019 Jan 14;15(1):e8438. doi: 10.15252/msb.20188438 (PMC6346213; doi:10.15252/msb.20188438)
Supplement: Supplementary file 7 — Dataset EV6 [file MSB-15-e8438-s007.zip › feature_plots_bioplex/Q9NVR5.pdf]

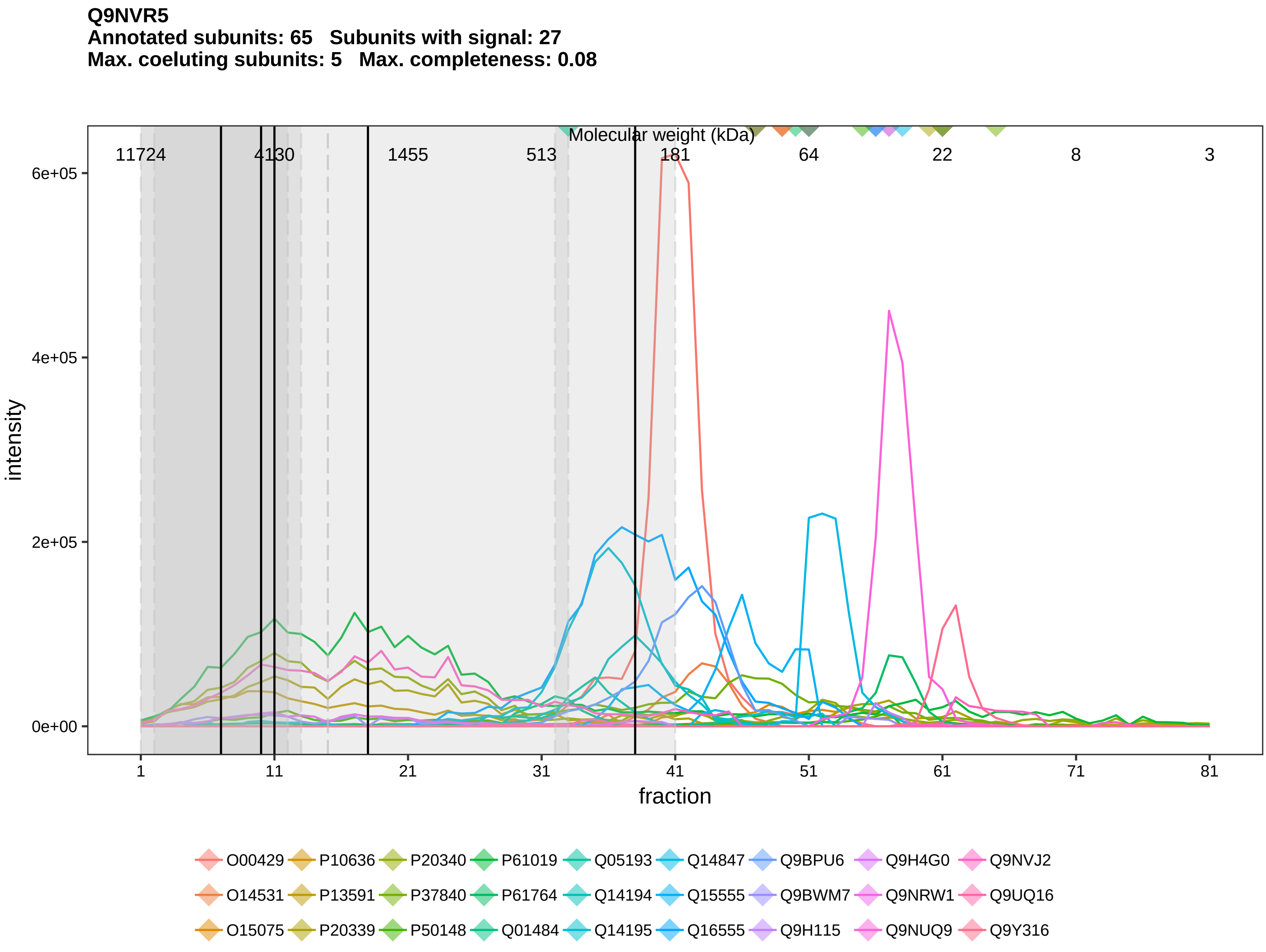
Q9NVR5
Annotated subunits: 65   Subunits with signal: 27
Max. coeluting subunits: 5   Max. completeness: 0.08
Molecular weight (kDa)
11724
4130
1455
513
181
64
22
8
3
intensity
6e+05
4e+05
2e+05
0e+00
fraction
1
11
21
31
41
51
61
71
81
O00429
P10636
P20340
P61019
Q05193
Q14847
Q9BPU6
Q9H4G0
Q9NVJ2
O14531
P13591
P37840
P61764
Q14194
Q15555
Q9BWM7
Q9NRW1
Q9UQ16
O15075
P20339
P50148
Q01484
Q14195
Q16555
Q9H115
Q9NUQ9
Q9Y316
